# Characterization of persistent post-traumatic headache and management strategies in adolescents and young adults following mild traumatic brain injury

**DOI:** 10.1038/s41598-022-05187-x

**Published:** 2022-02-09

**Authors:** Simple Futarmal Kothari, Peter Preben Eggertsen, Oana Veronica Frederiksen, Mille Moeller Thastum, Susanne Wulff Svendsen, Astrid Tuborgh, Erhard Trillingsgaard Næss-Schmidt, Charlotte Ulrikka Rask, Andreas Schröder, Helge Kasch, Jørgen Feldbæk Nielsen

**Affiliations:** 1 Hammel Neurorehabilitation Centre and University Research Clinic, Hammel, Denmark; 2 Section of Orofacial Pain and Jaw Function, Department of Dentistry and Oral Health, Aarhus University, Vennelyst Boulevard 9, 8000 Aarhus C, Denmark; 3 Scandinavian Center for Orofacial Neurosciences (SCON), Aarhus, Denmark; 4 Department of Occupational and Environmental Medicine, Bispebjerg and Frederiksberg Hospital, University of Copenhagen, Copenhagen, Denmark; 5 Department of Public Health, Section of Environmental Health, University of Copenhagen, Copenhagen, Denmark; 6 Department of Child and Adolescent Psychiatry, Research Unit, Aarhus University Hospital, Psychiatry, Aarhus, Denmark; 7 Department of Clinical Medicine, Aarhus University, Aarhus, Denmark; 8 The Research Clinic for Functional Disorders and Psychosomatics, Aarhus University Hospital, Aarhus, Denmark; 9 Department of Neurology, Regional Hospital of Viborg, Viborg, Denmark

**Keywords:** Medical research, Neurology, Signs and symptoms

## Abstract

Characteristics of persistent post-traumatic headache (PTH) in young individuals are poorly known leading to diagnostic problems and diverse management. We aimed to describe headache phenotypes and self-reported management strategies in young individuals with PTH following mild traumatic brain injury (mTBI). A comprehensive structured questionnaire was used to evaluate headache phenotypes/characteristics and management strategies to relieve headache in 107, 15–30-year-old individuals with PTH. Around 4 months post-injury, migraine-like headache in combination with tension-type like headache (40%) was the most commonly encountered headache phenotype followed by migraine-like headache (36%). Around 50% reported aura-like symptoms before/during the headache attack. Medication-overuse headache was diagnosed in 10%. Stress, sleep disturbances, and bright lights were the most common trigger factors. More than 80% reported that their headache was worsened by work-related activity and alleviated by rest/lying down. Simple analgesics were commonly used (88%) whereas prophylactic drugs were rarely used (5%). Bedrest and physiotherapy were also commonly used as management strategies by 56% and 34% of the participants, respectively. In conclusion, most young individuals with PTH after mTBI presented with combined migraine-like and tension-type-like headache followed by migraine-like headache, only. Preventive headache medication was rarely used, while simple analgesics and bedrest were commonly used for short-term headache relief.

## Introduction

The annual incidence of traumatic brain injury (TBI) is estimated at 50 million cases worldwide^1^. Mild traumatic brain injury (mTBI) accounts for 80–90% of these injuries^2^. Although most individuals with mTBI recover within the first few weeks, 10–15% experience persistent post-concussion symptoms (PCS) which often include post-traumatic headache (PTH), a secondary headache disorder^3^. Indistinct disease classification, poorly described clinical features and unclear pathophysiological mechanisms lead to diverse and sometimes complicated treatment regimens with unclear effects^4–7^. Thus, PTH remains a challenge to both the affected individuals and their relatives and health-care providers^5,8^.

According to the International Classification of Headache Disorders, 3rd edition (ICHD-3), onset of headache within 7 days of head trauma is defined as PTH attributed to TBI, and if the headache persists for more than 3 months after onset, it is classified as persistent PTH^9^. Although there are criteria for diagnosing persistent PTH, descriptions of the phenotypes of persistent PTH vary. Migraine/probable migraine appears to be the most common phenotype around 3 months^10,11^ and more than 1 year^12^ after mTBI in studies of civilian populations, and several months to years after mTBI in military populations^13,14^. However, studies conducted in a highly specialized headache clinic have found tension-type headache (TTH) to be the predominant PTH phenotype several years after mTBI^5,15^. In some cases, trigeminal autonomic cephalalgias-like (TACs-like) and cervicogenic phenotypes were described, and a number of cases have remained unclassifiable^10,13^.

Knowledge of persistent PTH phenotypes in adolescents and young adults is particularly limited, although the incidence of mTBI in this age group is high^16^. It is not known whether the characteristics of persistent PTH after mTBI differ between individuals who are seen in hospital in connection with the trauma and those individuals who only contact their GP later on. In addition, there are no evidence-based guidelines for the management of PTH, so the management seems to be mostly guided by protocols used for the treatment of the primary headache phenotype it resembles the most^17^. Only few studies have reported the management strategies of persistent PTH in young people^18–20^. Therefore, knowledge of the PTH phenotypes and the different management strategies in this group may be important for establishing an evidence-based management strategy for PTH in the future.

The aim of this study was to describe the headache phenotypes of persistent PTH approximately 4 months after mTBI along with self-reported management strategies in 15–30-year-old individuals.

## Methods

### Design and participants

**Figure 1:**
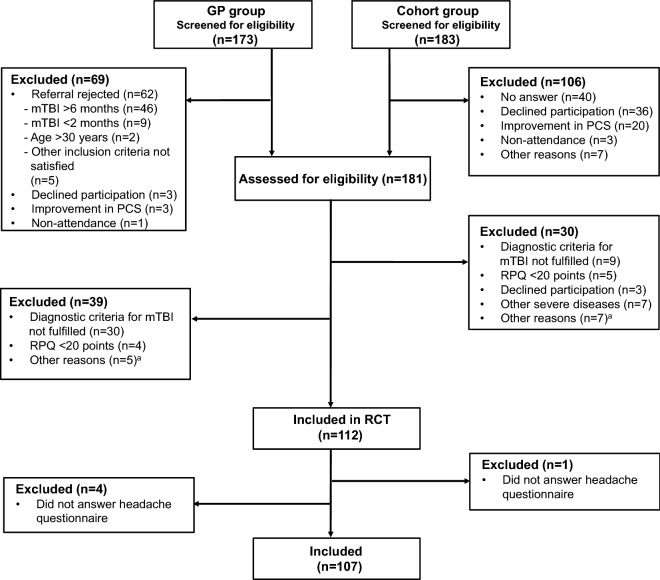
Flowchart of the study. All participants had persistent post-traumatic headache. ^a^Other reasons include: previous mTBI within the last 2 years leading to post-concussion symptoms lasting ≥ 3 months; signs of more severe brain injury; other severe psychiatric or somatic disease including current substance abuse; inability to communicate in Danish; not from Central Denmark Region. *GP* general practitioner, *mTBI* mild traumatic brain injury, *PCS* post-concussion symptoms, *RCT* randomized controlled trial, *RPQ* Rivermead Post-Concussion Symptoms Questionnaire.

This was a cross-sectional study using baseline descriptive data from a randomized controlled trial (RCT) conducted in Central Denmark Region testing the effect of GAIN (Get going After concussIoN), a novel treatment for persistent PCS^21^. Individuals with mTBI and high levels of PCS 2–6 months after injury (see below) were identified from two sources: (1) referrals from general practitioners (the GP group) and (2) a cohort study investigating the prognosis after mTBI (the cohort group)^21^. The GPs referred individuals with PCS, they considered to be in need of treatment (i.e., interfering with patients ‘everyday life). However, the GPs themselves did not screen for PCS, i.e., patients from GPs were not screened for PCS prior to the clinical assessment (baseline questionnaire). For the cohort study, all individuals between 15 and 30 years of age diagnosed with mTBI (N = 3623) at public hospitals in Central Denmark Region from 2013 to 2017 were identified in an administrative hospital register. However, on initial contact, nearly two-thirds of the individuals (N = 2188) did not respond. From March 2015 through September 2017, potential participants from both the groups were then scheduled for a thorough clinical assessment to determine eligibility for the RCT based on severity of overall level of PCS 2–6 months after diagnosis^21^. Although, the individuals were recruited based on PCS in general, headache was the most common symptom at baseline. Thus, all the included participants in RCT (n = 112) reported headache as a symptom (Fig. 1). Baseline questionnaire data for the present study was collected in connection with the clinical assessment, which was performed by a neurologist and a psychiatrist. The assessments consisted of a neurological examination and a standardized psychiatric interview to assess eligibility^21^. The baseline questionnaire included the 16-item Rivermead Post-Concussion Symptoms Questionnaire (RPQ) to measure the severity of PCS [range 0–64 (worst)]^22^ and a comprehensive, structured headache questionnaire (see below). The inclusion criteria were: (1) mTBI within the last 2 to 6 months according to the diagnostic criteria recommended by the WHO Task Force^23^, (2) 15–30 years old at the time of mTBI, (3) high levels of PCS defined as a score of ≥ 20 points on the RPQ, and (4) a direct contact between the head and an object. Although, we tried to rule out acceleration-deceleration traumas that may only have caused injury to the neck tissue, the included patients might have had some degree of concomitant whiplash^21^. Patients were excluded in case of (1) previous mTBI within the last 2 years leading to PCS lasting ≥ 3 months, (2) objective neurological findings from neurological examination indicating other neurological disease or more severe brain injury, (3) other severe psychiatric or somatic disease that would impede participation in the RCT, such as bipolar disorder, autism, psychotic disorder (life time), multiple sclerosis etc. as well as current substance abuse, (4) inability to communicate in Danish and (5) not from Central Denmark Region. RCT participants, who did not answer the headache questionnaire, were also excluded from the present study. All participants fulfilled the criteria for persistent PTH attributed to mTBI in accordance with the ICHD-3^9^, except for four individuals where the headache did not develop within 7 days following mTBI. However, the requirement of developing headache within 7 days is arbitrary and is not based on scientific evidence^24^. Moreover, out of these four individuals, two individuals reported of having no headache prior to the trauma indicating that the headache that developed post-trauma was attributed to mTBI. In the other two individuals, the headache frequency was only 1–14 headache days per year pre-trauma whereas post-trauma, they reported to have 8–31 headache days per month. Thus, indicating that the increase in the headache frequency was most likely due to mTBI. Therefore, we also included these four individuals in the study. All participants gave oral and written informed consent. The study was conducted in accordance with the Declaration of Helsinki, with subsequent revisions. The RCT study was approved by the Danish Data Protection Agency (no. 1-16-02-23-15) and by the Committee of Health Research Ethics of Central Denmark Region (no. 1-10-72-79-14).

### Demographic data and trauma-related characteristics

Questionnaire data was collected on demographic characteristics such as age at injury, sex, education, cohabitant status, and employment status, and on trauma-related characteristics in terms of time since trauma, total RPQ-score, RPQ-headache score [1 item, range 0–4 (worst)], and causes of trauma.

### Headache questionnaire

The headache questionnaire consisted of 27 questions covering the following: headache history (pre- and post-trauma), headache characteristics in terms of frequency, current and worst intensity during the last 2 weeks (measured using a visual analogue scale (VAS) ranging from 0 to 10 cm: mild (1–3), moderate (4–7) and severe (8–10)^25^), duration of headache attacks, location (unilateral, bilateral, and other), quality [feeling heaviness, pressing, throbbing, tightening, pulsating, stabbing and other (burning, aching, and pounding)], aura symptoms, autonomic symptoms and other accompanying symptoms (phonophobia, photophobia, dizziness, nausea, insomnia, feeling cold, sweating, tinnitus, feeling warmth, vomiting, diarrhea or constipation), trigger factors [stress (not further specified), bright lights, insomnia, neck pain, alcohol, skipped meals, prolonged sleep, and other (warmth, hormonal factors, perfume, and smoking)], worsening/alleviating factors (work, sudden movements, physical activity, head movements, standing up, chewing, intercourse, rest/lying down, and other), and pharmacological (paracetamol (acetaminophen), nonsteroidal anti-inflammatory drugs (NSAIDs), acetylsalicylic acid, codeine, tramadol, morphine, triptans, anticonvulsants, or antidepressants) and non-pharmacological management strategies (physiotherapy, heat/cold applications, exercise, bedrest, strength training, relaxation techniques, chiropractic, acupuncture, psychological treatment, or alternative treatment) to alleviate the headache and their effect. The effect of these management strategies on persistent PTH was measured in terms of reported percentage (0%, 25%, 50%, 75% and 100%) reduction in the headache intensity, where 0% = no effect, 25% = little effect, 50% = good effect, 75% = very good effect and 100% = headache free. Headache relief was defined as a reported reduction in headache intensity of at least 50%. The data regarding use of different management strategies for headache relief was collected as a part of the headache questionnaire at the time of enrolment, thus prior to the GAIN intervention.

The questionnaire also included the six-item Headache Impact Test (HIT-6)^26^. HIT-6 is a reliable and validated tool to measure the severity of headache and the impact that headache has on social functioning, role functioning, vitality, cognitive functioning, and psychological distress^26^. We used the recommended item response weights (never = 6, rarely = 8, sometimes = 10, very often = 11 and always = 13) and calculated the HIT-6 score as the sum of the six item response weights [range 36–78 (worst)].

### PTH phenotypes

Based on the responses from the headache questionnaire, PTH phenotypes were classified into one of the following primary headache types in accordance with ICHD-3^9^: (1) migraine-like, (2) TTH-like, (3) probable TACs-like (4) mixed (migraine-like and TTH-like), and 5) unclassifiable (when the headache characteristics did not fit into one of the just-mentioned categories). The classification was exhaustive and mutually exclusive. A headache was defined as migraine-like if it fulfilled the C and D criteria together with A and/or B for *1.1 Migraine without aura* in accordance with ICHD-3^9^. A TTH-like phenotype was defined as any headache that fulfilled criteria A–D for *2.1 Infrequent episodic tension-type headache*, *2.2 Frequent episodic tension-type headache*, *2.3 Chronic tension-type headache or 2.4 Probable tension-type headache*^9^. However, in accordance with the ICHD-3, if a participant fulfilled both a probable diagnosis and a definite diagnosis, it was coded as the latter. A TACs-like phenotype was defined as any headache that fulfilled the B and C criteria for *3.1 Cluster headache*, *3.2 Paroxysmal hemicrania*, and *3.3 Short-lasting unilateral neuralgiform headache attacks*, or criteria A–C for *3.4 Hemicrania continua*^9^. In addition to the above classification, probable medication overuse headache (MOH), a secondary headache, was assessed in accordance with ICHD-3^9^. Further, the headache questionnaire asked about the occurrence/development of aura before and/or during the headache attacks. An aura was defined as visual disturbances like blind spots, seeing flashing lights or bright spots, impaired vision/tunnel vision, sensory disturbances like tingling or feeling numbness in the face, hand/arm or any other part of the body and speech disturbances like altered speech. However, as information on duration of aura symptoms was lacking, it was not feasible to sub-classify migraine-like headache into migraine with and without aura.

### Statistical analysis

For categorical variables, the number and percentage of participants were provided per category, while the mean and standard deviation (SD) or median and interquartile range (IQR) were given for continuous and discrete variables. The analyses were carried out using Statistical Package for the Social Sciences (SPSS), version 24.0 (Armonk, New York: IBM Corp.).

## Results

### Demographics and trauma-related characteristics

Figure 1 displays the flowchart showing inclusion and exclusion of the participants. The mean time from trauma to assessment was 120 days (SD 35.7). The demographics of the 107 participants are presented in Table 1. The majority of the participants were females, had graduated from high school, were living with friends or partner, were on full-time or part-time sick leave, and had sustained an injury in traffic accident. Of the 107 participants, 83 (78%) were adults and 24 (22%) were adolescents. A greater percentage of the GP group (41%) was on full-time sick leave than the cohort group (13%). The most common cause of trauma in the GP group was fall (30%), whereas, in the cohort group, it was traffic accident (44%). The range of RPQ-headache was 2–4 in both the groups. The average HIT-6 score was 64.1 (7.8).

**Table 1:** Demographic data and trauma-related characteristics. Numbers in cells are n (%) unless otherwise specified.

	GP group n = 61	Cohort group n = 46	Total N = 107
Age, years, mean (SD)	23.1 (4.7)	22.4 (3.7)	22.8 (4.3)
**Sex**			
Female	45 (74)	39 (85)	84 (79)
Male	16 (26)	7 (15)	23 (21)
**Education**			
Primary school	21 (34)	18 (39)	39 (36)
Secondary or high school	22 (36)	21 (46)	43 (40)
Higher education	13 (21)	7 (15)	20 (19)
Unknown	5 (8)	0 (0)	5 (5)
**Cohabitant status**			
Living alone	3 (5)	6 (13)	9 (8)
Living with parents	18 (30)	16 (35)	35 (33)
Living with friends/partner	26 (43)	15 (33)	40 (37)
Unknown	14 (23)	9 (20)	23 (22)
**Employment status**			
Full-time sick leave	25 (41)	6 (13)	31 (29)
Part-time sick leave	19 (31)	14 (30)	33 (31)
Full-time employment/education	13 (21)	18 (39)	31 (29)
Part-time employment/education	0 (0)	3 (7)	3 (3)
Unknown	2 (3)	2 (4)	4 (4)
Other	2 (3)	3 (7)	5 (5)
**Trauma-related characteristics**			
Time since trauma, days, median (IQR)	111 (60)	118 (41)	117 (47)
Total RPQ-score**,** mean (SD)	37.6 (8.4)	37.6 (8.0)	37.6 (8.2)
RPQ-headache^a^**,** mean (SD)	3.7 (0.5)	3.2 (0.6)	3.5 (0.6)
HIT-6, mean (SD)	65.2 (4.5)	63.3 (4.6)	64.1 (7.8)
**Causes of trauma**			
Traffic accident	11 (18)	20 (44)	31 (29)
Sports	14 (23)	12 (26)	26 (24)
Hit by object	15 (25)	8 (17)	23 (22)
Fall or assault*	21 (34)	6 (13)	27 (25)

*A total of 4 individuals reported assault. a The range of RPQ-headache was 2–4 in both the groups. The percentages do not always add up to 100% due to rounding.

*GP* general practitioner, *HIT* headache impact test [range 36–78 (worst)], *IQR* interquartile range, *RPQ* Rivermead Post-Concussion Symptoms Questionnaire [range 0–64 (worst)], *SD* standard deviation.

### Headache characteristics including PTH phenotypes

**Figure 2:**
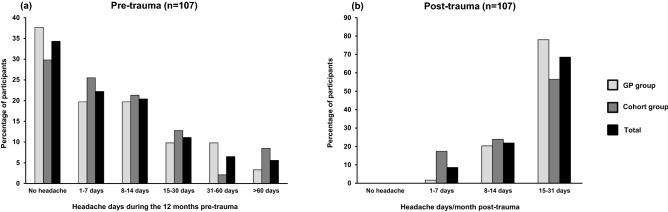
(**a**) Headache days during the 12 months pre-trauma; (**b**) headache days/month post-trauma. *GP* general practitioner.

Figure 2a shows the distribution of headache days during the 12 months pre-trauma. Overall, 44% (47/107) reported having headache before trauma (disregarding headache only for 1–7 days during the preceding year). The pre-trauma headache was unclassifiable in 59% (28/47) due to limited information on headache characteristics but could be classified as probable TTH in 28% (13/47), and as probable migraine in 13% (6/47). Figure 2b shows the distribution of headache days/month post-trauma. A total of 69% reported 15–31 headache days/month. Among participants from the GP group, 78% reported 15–31 headache days and 57% in the cohort group. The opposite was true for 1–7 headache days, where the cohort group dominated (17%) compared to the GP group (2%).

The PTH phenotypes are presented in Table 2. The most common phenotype was mixed (migraine-like and tension-type like) followed by migraine-like headache, only. Therefore, more than two-third [(76%; migraine-like only (36%) plus mixed (40%)] represented with migraine-like complaints and more than half [(55%; TTH-like only (15%) plus mixed (40%)] of the patients with tension-type like headache. Probable MOH was diagnosed in 10% of the participants. As shown in Table 3, the mean current intensity of headache was moderate and the mean worst intensity during the last 2 weeks was severe. Headache was most commonly reported to be unilateral, with a preponderance to the frontal region (62%) or the occipital region (13%). The most prevalent headache qualities were “feeling heaviness” and “pressing”. Aura symptoms were experienced by around half of the participants. Phonophobia was the most common accompanying symptom and stress was the most common trigger of PTH. More than 50% of the participants reported that work activity, sudden movements, and physical activity worsened the headache, whereas 84% reported that rest/lying down alleviated it (Table 3).

**Table 2:** Headache phenotypes of persistent post-traumatic headache* approximately 4 months after mild traumatic brain injury.

Headache phenotype	GP group n = 61	Cohort group n = 46	Total N = 107
Migraine-like	21 (34)	17 (37)	38 (36)
TTH-like	10 (16)	6 (13)	16 (15)
Mixed (migraine-like and TTH-like)	24 (39)	19 (41)	43 (40)
TACs-like	4 (7)	3 (7)	7 (7)
Unclassifiable	2 (3)	1 (2)	3 (3)

Numbers in cells are n (%).

*Classified according to the International Classification of Headache Disorders, 3rd edition. The percentages do not always add up to 100% due to rounding.

*GP* general practitioner, *TACs* trigeminal autonomic cephalalgias, *TTH* tension-type headache.

**Table 3:** Headache characteristics approximately 4 months after mild traumatic brain injury.

**Headache intensity**				
Current VAS, mean (SD)	5.2 (2.1)			
Worst VAS during the last 2 weeks, mean (SD)	8.2 (1.4)			
**Headache localization**	**n (%)**	**95% CI**		
Unilateral	53 (49.5)	39.7–59.4		
Bilateral	46 (43.0)	33.5–52.9		
Unclassifiable/unknown	8 (7.5)	3.3–14.2		
**Headache quality**				
Feeling heaviness	82 (76.6)	67.5–84.3		
Pressing	81 (75.7)	66.5–83.5		
Throbbing	68 (63.5)	53.7–72.6		
Tightening	66 (61.7)	51.8–70.9		
Feeling heaviness and pressing	64 (59.8)	49.9–69.2		
Feeling heaviness and throbbing	58 (54.2)	44.3–63.9		
Throbbing and pressing	50 (47.0)	37.0–56.6		
Pulsating	36 (33.6)	24.8–43.4		
Stabbing	36 (33.6)	24.8–43.4		
Other	≤ 15 (14.0)	8.1–22.1		
**Aura symptoms**	53 (49.5)	39.7–59.4		
Visual	15 (14.0)	8.1–22.1		
Visual and sensory	9 (8.4)	3.9–15.4		
Sensory	7 (6.5)	2.7–13.0		
Visual and speech	7 (6.5)	2.7–13.0		
Speech	3 (2.8)	0.5–7.9		
Sensory and speech	3 (2.8)	0.5–7.9		
Visual, sensory and speech	3 (2.8)	0.5–7.9		
Other	6 (5.6)	2.1–11.8		
**Accompanying symptoms**				
Phonophobia	92 (86.0)	77.9–91.9		
Photophobia	87 (81.3)	72.6–88.2		
Dizziness	66 (61.7)	51.8–70.9		
Nausea	52 (48.6)	38.8–58.5		
Insomnia	33 (30.8)	22.3–40.5		
Feeling cold	28 (26.2)	18.1–35.6		
Sweating	22 (20.6)	13.4–29.5		
Tinnitus	20 (18.7)	11.8–27.4		
Feeling warmth	20 (18.7)	11.8–27.4		
Vomiting	7 (6.5)	2.7–13.0		
Diarrhea or constipation	*	*		
**Trigger factors**				
Stress	78 (72.9)	63.4–81.0		
Bright lights	67 (62.6)	52.7–71.8		
Insomnia	66 (61.7)	51.8–70.9		
Neck pain	60 (56.1)	46.1–65.7		
Alcohol	27 (25.2)	17.3–34.5		
Skipped meals	20 (18.7)	11.8–27.4		
Prolonged sleep	18 (16.8)	10.3–25.3		
Other	≤ 15 (14.0)	8.1–22.1		
**Worsening/alleviating factors**	**Worsens headache**		**Alleviates headache**	
	**n (%)**	**95% CI**	**n (%)**	**95% CI**
Work	92 (86.0)	77.9–91.9	3 (2.8)	0.5–7.9
Sudden movements	75 (70.1)	60.5–78.6	*	*
Physical activity	69 (64.5)	54.6–73.5	15 (14.0)	8.1–22.1
Head movements	63 (58.9)	49.0–68.3	7 (6.5)	2.7–13.0
Standing up	43 (40.2)	30.8–50.1	7 (6.5)	2.7–13.0
Chewing	20 (18.7)	11.8–27.4	*	
Intercourse	15 (14.0)	8.1–22.1	11 (10.3)	5.2–17.7
Rest/lying down	*	*	90 (84.1)	75.8–90.5

*CI* confidence interval, *SD* standard deviation, *VAS* visual analogue scale.

*< 3 individuals.

### Management strategies

**Figure 3:**
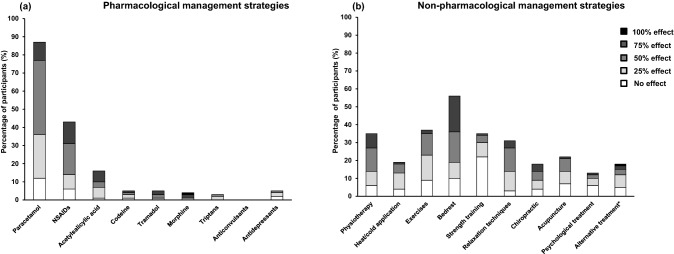
Management strategies to relieve persistent post-traumatic headache (**a**) pharmacological and (**b**) non-pharmacological. *The most common alternative treatment was craniosacral therapy. 25%, 50%, 75% and 100% effect indicates reported reduction in headache severity/intensity by 25% (little effect), 50% (good effect), 75% (very good effect) and 100% (headache free), respectively. *NSAIDs *nonsteroidal anti-inflammatory drugs.

As shown in Fig. 3a,b, both pharmacological and non-pharmacological management strategies were frequently used (pharmacological: 93%; non-pharmacological: 79%). Acute medications such as paracetamol (88%) and NSAIDs (43%) prevailed (Fig. 3a). Paracetamol relieved headache in 46% (44/95) and NSAIDs in 39% (18/46) of the participants using these medications. Only 4% (4/107) of the participants were prescribed triptans and prophylactic drugs (anticonvulsants: 0% and antidepressants: 5%) were also rarely prescribed. The most common non-pharmacological management strategies were bedrest (56%), exercises (37%), physiotherapy (34%), and relaxation techniques (31%) (Fig. 3b). Among the participants who used bedrest, headache was reported to be relieved in 65% (39/60). Physiotherapy and relaxation techniques relieved headache in 60% (22/37) and 55% (18/33), respectively. The most common combination was physiotherapy and exercises (27%) which relieved headache in 34% (10/29).

## Discussion

This study comprehensively describes the headache characteristics in individuals aged 15–30 years with persistent PTH attributed to mTBI. Most participants reported migraine-like headache (typically in combination with TTH-like headache) and reported 15–30 headache days per month. Stress was reported as the most common trigger factor. In more than 80% of participants, headaches were aggravated by work and relieved by rest/lying down. In addition, simple analgesics were commonly used whereas specific anti-migraine and prophylactic drugs were rarely prescribed.

### Headache characteristics including PTH phenotypes

In the current study, the headache was reported with a typical frequency of 15–30 headache days/month consistent with previous findings^5,12,14,15^. In particular, our results can be compared to a recent study by Ashina et al., which also applied the ICHD-3 criteria to characterize persistent PTH and had a majority of female participants, although in that study the average time since trauma was around 4 years^12^. Our findings of 15–30 headache days/month, moderate to severe headache intensity, and the frontal region as the most common location are consistent with that study^12^, as opposed to our finding that headache was more often unilateral (50%) than bilateral (43%). This difference does not seem to be due to the inclusion of adolescents in the present study, as migraine often occurs bilaterally in adolescents and unilaterally in adults^9^. In general, the findings regarding localization of PTH have been inconsistent, which may be related to trauma mechanisms^10–12^. Previous studies have used the term pressing to characterise headache quality^11,12^. Most participants in the study by Ashina et al.^12^ experienced throbbing and pressing headache (combined), which was also prevalent in our study. However, most of our participants reported “feeling heaviness” as a headache quality, either alone or in combination with other qualities. We added the term “feeling heaviness” to the qualities mentioned in ICHD-3, which is why this term has not been used in previous studies of PTH.

In the present study, the most common headache phenotype was migraine-like (primarily in combination with TTH-like), which is in agreement with most previous studies^10,12,27,28^, apart from a few studies conducted in tertiary settings^5,15^. Also consistent with a recent study^12^, stress, sleep disturbances, and bright light were common triggers, i.e. factors which are also reported to trigger migraine^29^. Additionally, photophobia, phonophobia, and nausea were symptoms that were commonly associated with PTH, and work, sudden movements, and physical activity typically worsened the headache which is also a characteristic of migraine^9^. This indicates that migraine and persistent PTH overlap and may share a common pathophysiology^24^.

In the present study, aura symptoms were reported by half of the participants. The high prevalence may be due to the young age of the study population, as aura symptoms in migraine patients seem to decrease with age^30,31^. However, previous studies that have enquired into aura in PTH patients reported that < 15% had aura or had migraine with aura^5,12,14^. In the present study, where the subjects were assessed already after a median of 4 months, the aura symptoms might have occurred as a part of mTBI itself caused by cortical spreading depression^24^. Longitudinal studies in individuals with incident mTBI are needed to evaluate the development of aura over time. To further support this finding, it would have been of interest to evaluate if the patients with aura symptoms had migraine headache previous to the mTBI. However, the limited information that was obtained on clinical characteristics of pre-trauma headache did not allow us to classify the pre-trauma headaches into different types in accordance with ICHD-3^9^.

### Management strategies

Current pharmacological management strategies for PTH are based on acute and preventive medications used for primary headache disorders^17^. In the present study, a majority of participants used acute treatment with simple analgesics which often provided relief from headache. However, the development of MOH should be considered, as was also seen in the present study. Triptans and prophylactic drugs were rarely used, probably because our study population had a relatively recent mTBI as compared to patients treated in specialized headache centres, where prophylactic drugs and triptans have been reported to be commonly used^12,27^. Nevertheless, the use of these drugs has been reported to be less efficacious for treatment of persistent PTH^4,12,17^.

Participants also opted for a range of non-pharmacological treatments such as bedrest and relaxation techniques. Although strategies such as bedrest may provide short-term relief from the headache as reported by participants in the present study, there is a risk that it may lead to disability and loss of function in the long-term. An approach in which individuals are encouraged to gradually be more active may prove more helpful^32^. Two recent RCTs employing interdisciplinary interventions encouraging active management strategies including gradual return to activity have shown significant reductions in the level of persistent PCS^21,33^, including the RCT that provided baseline data for the present study^21^. The specific effect of the GAIN intervention of the RCT providing baseline data for this study remains to be evaluated on headache intensity and frequency^21^. Further, due to the heterogeneity of the data on the treatment strategies used, we were unable to assess the distribution of PTH phenotypes across different treatment strategies used.

### The GP group versus the cohort group

The GP group was more likely to have full-time sick leave and had more headache days/month than the cohort group. Although, the reason for this is not known. An inverse relationship has been suggested between the severity of PCS including PTH and the severity of the head trauma^34^. Therefore, it may be speculated that the fall injuries causing mTBI in majority of the individuals in GP group were less severe traumas than the injuries caused by the traffic accidents suffered by the majority of the cohort group. Furthermore, in a biopsychosocial understanding, the majority of individuals with persistent PCS also suffer from psychosocial difficulties/abnormalities including psychological distress^35,36^. Thus, they seek help from the GP as GP acts as a gatekeeper before further referral, which leads to more sick leave. In Denmark, it is usually the GP who prescribes the sick leave^37^. In accordance with this hypothesis, a study reported that there was a low frequency of sick leave in individuals with mTBI admitted to a hospital^38^. The burden of headache, as measured by the total RPQ-score, the RPQ-headache score, and HIT-6, and the distribution of PTH phenotypes were similar in both groups.

### Strengths and limitations

The present study has several strengths. The study population was confined to young individuals with mTBI, who usually have less/no comorbidities. Furthermore, we excluded individuals with severe psychological/somatic disease, which could have influenced the characteristics of persistent PTH. The study also allowed for the comparison of participants from primary care and public hospitals. The study also has some limitations. During the enrolment period, nearly two-thirds of individuals with mTBI did not respond to initial contact, which otherwise would have resulted in inclusion of large number of participants. This relatively small response on initial contact might have led to selection bias. We applied a headache questionnaire instead of a validated headache diary which may have resulted in recall bias. However, we believe that this was a minor problem as the data was collected around 4 months after mTBI, unlike other studies that have assessed PTH about 4 years after mTBI^12^. We chose to use the questionnaire because we thought that filling in a headache diary might be too challenging for our young participants which could have led to lower participation or more missing information. Further, it may be a limitation that we were not able to include the individuals with mTBI who presented with headache as the only symptom because they would not have fulfilled the inclusion criteria of having a RPQ score > 20. However, mTBI individuals with persistent PCS usually suffer from a range of different symptoms rather than a single symptom^39^. Therefore, it is unlikely that we missed many patients suffering from headache only 2–6 months post-trauma. In the present study, we were unable to assess the relationship between headache characteristics before and after trauma because little information was available on headache characteristics before trauma. Previously, a few studies have shown that history of pre-trauma headache is a predictor for headache following TBI^40–42^. In particular, a robust association has been shown between pre-trauma migraine-like headache and headache severity following moderate and severe TBI^41^. Furthermore, we did not include control groups of individuals with mTBI without PCS and healthy age-matched individuals, in order to compare the headache characteristics between these control groups and individuals with persistent PTH both pre- and post-trauma, which could have provided information on pre-trauma headache as a predisposing factor of PTH. Nevertheless, the purpose of the study was to describe the PTH characteristics and not to evaluate the relationship between the pre- and post-trauma headache. The fact that almost 80% of the participants were women indicates selection bias. This may be related to the fact that women reported more symptoms and therefore more easily met the inclusion criterion of an RPQ score ≥ 20 points^43^. Furthermore, although males experience mTBI more frequently than females, PTH incidence is reported to be greater in females than in males^39^. Weaker neck musculature in females compared to males may also be a contributing factor to higher PTH prevalence in females^44,45^. Another explanation might be that women are more willing to seek treatment and participate in scientific studies than men^46^. This means that our results may not reflect the characteristics of PTH in men as accurately as in women. Further, because of the small proportion of male participants compared with female participants, we were unable to evaluate the differences in PTH phenotype, other characteristics, and management strategies used between the two sexes. For the same reason, no analysis was performed to examine the differences in PTH characteristics and management strategies between the adolescents and adults, as the proportion of adolescents (22%) was small compared to the adults (78%). Nevertheless, the results regarding PTH phenotypes and management should be generalizable to other populations of young individuals with persistent PTH around 4 months after mTBI.

The clinical implication of this study is that it provides information on the common headache features and associated symptoms that occur in young individuals with mTBI having persistent PTH. This will increase clinicians' awareness of PTH symptomatology and facilitate more informed clinical decision making. Further, 10% of the individuals with PTH also developed probable MOH, further complicating the management of persistent PTH. Thus, it is important that the clinicians also diagnose MOH in these patients and provide appropriate treatment.

## Conclusions

Around 4 months post-trauma, a migraine-like headache was the most common phenotype, typically in combination with a TTH-like headache. Aura symptoms were frequently reported. Pharmacological treatment with simple analgesics and non-pharmacological treatment in the form of bedrest were commonly used for short-term headache relief.

## Data Availability

The datasets generated during and/or analysed during the current study are available from the corresponding author on reasonable request.
